# Behavioral snake mimicry in breeding tits

**DOI:** 10.1093/cz/zoaa028

**Published:** 2020-06-11

**Authors:** Anders Pape Møller, Einar Flensted-Jensen, Wei Liang

**Affiliations:** 1 Laboratoire d’Ecologie, Systématique et Evolution, CNRS UMR 8079, Université-Paris-Sud, Saclay, Bâtiment 362, F-91405, Orsay Cedex, France; 2 Cypresvej 1, Brønderslev, DK-9700, Denmark; 3 Ministry of Education Key Laboratory for Ecology of Tropical Islands, College of Life Sciences, Hainan Normal University, Haikou, 571158, China

**Keywords:** Batesian mimicry, frequency-dependent selection, hissing display, personality, tits

## Abstract

Many animals mimic the behavior or the appearance of venomous snakes. When humans or other potential predators place their hand near the nest of tits belonging to the family Paridae (and a few other species), the incubating female performs a hissing display that mimics the inhalation hiss of a viper or another snake. They hiss vigorously while lunging their head forward and shaking their wings and tail, repeating this behavior several times. The structure of the hiss in tits is similar to that of the inhalation hiss of a snake, providing evidence of significant convergence of the mimic toward the model. The behavior of individual females is repeatable among trials. Individuals that flew away from their nest box only performed the hissing display on 6% of later trials, when present at their box, whereas individuals that did not fly away hissed on 28% of occasions, consistent with great tits *Parus major* either cautiously flying away or staying put on their nest while actively defending it. Individuals that flew away produced fewer chicks than individuals that stayed and hissed. The hissing display was more common when snakes were more abundant: 1) When breeding late during the season; 2) when breeding at sites with more snakes; and 3) when breeding in subtropical and tropical China with a higher abundance of snakes than in Denmark with a lower abundance. The frequency of nest predation was higher in sites with no snakes, and the frequency of predation increased with decreasing frequency of hissing display. These findings are consistent with expectations for frequency-dependent selection acting on snake mimicry.

Batesian mimicry occurs when an undefended species mimics a defended model that is either toxic or dangerous, and its efficiency relies on confusion of the mimic with the model (Bates 1862; Ruxton et al. 2004). Rare variants relative to the abundance of models have a disproportionate advantage in terms of elevated survival resulting in stable polymorphisms (Mallet and Joron 1999). Predator receivers have been assumed to gain information only from direct experience with the dangerous model (Speed and Turner 1999; Ruxton et al. 2004), although that cannot always be the case because death would then almost invariably result from encounters with venomous snakes. Snakes are likely to have converged on a common warning display to improve defense in terms of Müllerian mimicry. This may have facilitated the common evolution of inherited snake recognition mechanisms in most animals (Wilson 1975) that even seem to have an evolved neurobiological basis (Le et al. 2013).

Snakes are often venomous and hence constitute prime examples of models and mimics. Coral snakes Elapidae are highly venomous and several taxa of nonvenomous snakes have independently evolved a strikingly similar coloration and color pattern (Smith 1975, 1977; Pfennig et al. 2001). Several species of insects show snake mimicry with their body resembling that of the head of a snake (Wickler 1968). An example of a case of behavioral mimicry is the wryneck *Jynx torquilla* that twists and turns its head and neck in a manner similar to the movement of the head of a snake when captured by a human or another potential predator (Steinfatt 1941; Ruge 1971). Likewise, all vipers produce inhalation hissing sounds with a frequency ranging from 40 to 12,000 Hz (Aubret and Mangin 2014), and many species of birds perform a hissing display that closely mimics the sound of such a snake when confronted by a human or another potential predator (Bowles 1909; Sherman 1910; Coward 1920; Pickens 1928; Jourdain 1929; Burleigh 1930; Cox 1930; Grinnell et al. 1930; Jouard 1932; Odum 1941; Allen 1943; Bent 1946; Brooksbank 1949; Dixon 1949; Hinde 1952; Löhrl 1964; Gompertz 1967; Smith 1975, 1977; Czaplicki et al. 1976; Dixon 1983; Klump and Shalter 1984; Apel and Weise 1986; Krams et al. 2014).

The hissing display of an incubating or a brooding female cavity-nesting bird is a reaction to a nest intruder (Krams et al. 2014; Dutour et al. 2020), and the bird gives the display even before the lid of the nest box has been opened. The head is raised to ca. 60° above horizontal, with the white cheek patches ruffled and the crown feathers sleeked, the eyes “bulging,” the wings raised, the bird rises on its tarsi and utters an explosive hiss as the head is thrust forward like a snake, whereas at the same time the wings are brought sharply down and often strike against the sides of the nest cavity to make a booming sound, and whereas the mandibles are snapped shut at the end of the hiss (Hinde 1952; Gompertz 1967). Simultaneously the tail is fanned and the outermost white tail feathers are moved and clearly visible as if the bird is attracting attention to this part of the body. The hissing display is used when a potential nest predator arrives at and enters the nest cavity, and most predators subsequently disappear following the encounter resulting in elevated probability of survival by the hissing bird (Krams et al. 2014).

The hiss of a tit is remarkably similar to the hiss of a snake (Dutour et al. 2020). Different snakes show a high degree of acoustic similarity of their hiss, and interestingly this sound almost approaches the levels determined for white noise (Young et al. 1999; Young 2003; Aubret and Mangin 2014). Thus, there is a low level of acoustic specialization in the sounds produced by snakes, providing an efficient common warning display to improve defense as in Müllerian mimics. The high degree of similarity in snake hisses makes this an ideal model for the development of mimicry by birds. Indeed, sonograms of snake hisses and hisses by great tits *Parus major* are strikingly similar by consisting of highly repeated syllables of similar duration and frequency (Young et al. 1999, p. 2285).

The objectives of this study were to test for functional explanations of the occurrence of the hissing display in tits as an example of behavioral mimicry. We recorded whether tits gave the hissing display when checking nest boxes put up for breeding birds. These data were used to test 1) if the hissing display was repeatable among trails. This would be a requirement not only for the evolution of mimicry, but also for the evolution of different levels of mimicry in different populations. Next, we tested 2) whether tits that flew away from their box or stayed put differed in frequency of hissing display as expected if the 2 kinds of behavior constituted different personalities. We expected that individuals that stayed put hissed at a higher frequency than individuals that readily flew away. Furthermore, we tested 3) if the frequency of the hissing display increased with the abundance of snakes, as expected from frequency-dependent selection. We determined whether the relative frequency of hissing display in different study sites increased with the abundance of snakes. We also compared the frequency of hissing display in Denmark, where there is a low abundance and diversity of snakes with the frequency of the hissing display in subtropical and tropical China where many kinds of snakes are abundant. Finally, we tested 4) if the frequency of predation on nests was inversely related to the abundance of snakes, and whether a lower rate of predation occurred in sites with a higher frequency of hissing display.

## Materials and Methods

### Study areas

**Figure 1.**
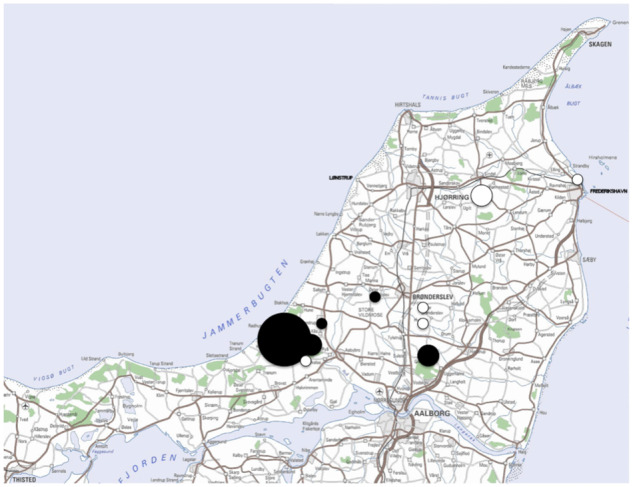
Location of the 10 study sites in Northern Denmark with the size of circles representing the number of nest boxes, black symbols reflecting sites with adders, and white symbols reflecting sites without adders.

We studied cavity-nesting passerines in nest boxes in 10 forests and plantations in Northern Jutland, Denmark, during March–July 1972, 2012, and 2013 with each site being studied in a single year (Figure 1). The study sites were located at distances of 5–70 km apart. The 10 sites were Tranum (57°58′N, 9°20′E), 224 boxes, Grishøjgård (57°15′N, 9°52′E), 13 boxes, Ulveskov (57°49′N, 9°23′E), 5 boxes, Ø. Brønderslev (57°15′N, 9°59′E), 6 boxes, Børglum Klosterskov (57°40′N, 10°22′E), 21 boxes, Knivholt (57°45′N, 10°48′E), 11 boxes, Kraghede (57°12′N, 10°00′E), 8 boxes, Hammer Bakker (57°53′N, 10°00′E), 38 boxes, Moseby (57°18′N, 9°65′E), 45 boxes, and Pandrup (57°13′N, 9°40′E), 14 boxes. The study sites varied in habitat from coniferous plantations (Tranum), over urban habitats with trees (Ø. Brønderslev) to mature deciduous forests dominated by beech *Fagus sylvatica* and conifers (Børglum Klosterskov)*.* All nest boxes were of a similar size and they were all situated at a height of 1–1.5 m along roads to facilitate nest checks.

The study sites in Southern and Central China were conducted during March–June 2013, at Diaoluoshan National Nature Reserve (18°40′N, 109°55′E), Hainan, which is covered with tropical forests, and at Dongzhai National Nature Reserve (32°15′N, 114°25′E), Henan, an evergreen broadleaf forest between subtropical and temperate zones (see Yang et al. 2012 for detailed descriptions of the study sites). All nest boxes were made by wood and of a similar size (35 cm in height and 11 cm in width and depth, with an entrance hole with a diameter of 4 cm). They were all situated at a height of 4–5 m along roads near forest edges. Because snakes or mammals depredated nests in 2012, the poles were provided with a plastic cover in 2013 to prevent all access to nests by nest predators. This was also the reason why only 14 great tit nests were tested for hissing behavior.

In Denmark, 72 boxes were occupied in 1972 and 313 in 2012–2013. In Denmark, 147 adults and 125 yearlings were tested for hiss calls, 41 could not be sexes.

### Recording life history traits

We visited nest boxes at 10-day intervals and recorded nest contents. The time of start of egg laying was derived from the number of eggs, assuming that 1 egg was laid daily. Clutch size was the maximum number of eggs present in a nest, whereas brood size at hatching was the number of nestlings present at the first visit and brood size at fledging the number of nestlings present at the last visit minus any dead nestlings left in the nest on the subsequent visit. Complete breeding failure occurred when no nestlings fledged. Nest predation occurred during the incubation and the nestling period, and we only recorded 2 cases of predation on adults.

### Age of breeding birds

We attempted to capture all females on the nest when visiting boxes following the recording of hissing display to avoid interference between capture and behavior. We aged great and blue tits *Cyanistes caeruleus* by relying on the coloration of the wing coverts that are pale in yearlings, but brightly colored in adults (Svensson 2006). Other species could not be aged, accounting for the smaller sample size in tests of aged individuals. The minimum age of birds was determined according to the year when first captured or when ringed as a nestling.

### Recording hissing display

When we visited nest boxes, we opened the lid of the nest box and placed a hand on the rim. The response of the incubating females was recorded as 1) “flew away” when the bird left the nest box without any display; 2) it did not react; or 3) it gave a hissing display by calling and attacking the hand. These responses were transformed into 2 categorical variables scored as 0 or 1 for staying put or flying away, and 0 or 1 when the bird did not give a hissing display or it did. This information was recorded for 423 visits to 224 occupied boxes in 2013 (many boxes were visited more than once). We recorded the presence or absence of hissing behavior on 1–3 occasions to avoid habituation. An example of a hissing display can be found in the attached video (Supplementary Video S1).

### Abundance of snakes

This part of the study on the abundance of snakes was based on 385 nest boxes distributed among 10 study sites during 1972 and 2012–2013. The only species of snake in the Danish study sites is the common adder *Vipera berus*. We recorded all observations of adders while checking the nest boxes at the 10 study sites, in total 26 adders were recorded during 270 h of fieldwork. The 5 sites with adders present and the 5 sites without adders were identical to the distribution according to the information in the Danish atlas of amphibians and reptiles (Fog 2001). Nest boxes were checked by E.F.-J. and A.P.M. We cannot exclude that the same individual adder was observed more than once, but even if that was the case, differences in the frequency of encounters with adders should be consistent among sites. Since each nest box was visited 3 times during the breeding season with a total observation effort of 270 h, we used the number of adders observed per hour of fieldwork as a relative estimate of the abundance of adders. Although several sites did not have any adders, others varied considerably in their abundance of adders from rare to very common. The abundance of adders in 2012 and 2013 for the same 10 sites was highly repeatable (*F *=* *40.21, df *=* 9, 10, *P *<* *0.001; *R *=* *0.95, SE = 0.04).

### Statistical analyses

We estimated repeatability of hissing behavior using the intra-class correlation coefficient (Falconer and Mackay 1996). This estimate also provides an upper limit to the heritability of the trait (Falconer and Mackay 1996). We used generalized linear models with a binomial error distribution to test predictions. In the first test, we used hissing display (or not) as the response variable and the dichotomous variable “fly away” or “not” as a predictor. In the second test, we used hissing display (or not) as the response variable and stage in the breeding cycle, age, and life history variables as predictors. For the third prediction, we used hissing display (or not) in the different sites as the response variable and the abundance of snakes as a continuous predictor variable. In addition, we used hissing display as the response variable and laying date as a continuous predictor variable with species as a random effect to account for differences in sample size among species. Furthermore, we used hissing display or not as the response variable and country as a dichotomous predictor. For the 4th prediction, we used the frequency of nest predation as a continuous response variable and the abundance of snakes as a predictor. Finally, we repeated this test by inclusion of the frequency of hissing as a continuous predictor. We used female identity as a random effect in these models to account for variation in number of observations per female (1–3 observations).

We weighted the analyses by sample size to account for variation in sample sizes and hence the precision of estimates among species and study sites (Garamszegi and Møller 2010, 2011, 2012). Most statistical analyses assume that data points provide equally precise information about the deterministic part of total process variation, that is, the standard deviation of the error term is constant across all values of the predictor variable (Sokal and Rohlf 1995). Bias due to variation in sample size can be a major problem in statistical analyses (Garamszegi and Møller 2010, 2011. If this assumption of even sampling effort is violated, weighting each observation by sampling effort allows for the rigorous use of all data, giving each datum a weight that reflects its degree of precision due to sampling effort (Draper and Smith 1981; Sokal and Rohlf 1995; Neter et al. 1996). This procedure also allows both sites with few and many nest boxes to be included. All statistical analyses were made using JMP (SAS 2012).

## Results

### Occurrence of hissing displays

Hissing behavior occurred in 27% of 313 individuals, 95% confidence interval 22–32%. The probability of hissing behavior in 125 yearlings was 34% (variance 23), but 23% (variance 18) in 147 older individuals. Both the mean value and the variance were significantly larger in yearlings than in older birds (Welch Anova for unequal variances: *F*_1,251_ = 3.63, *P *=* *0.05; Levene’s test for equal variances: *F*_1,270_ = 14.20, *P *=* *0.0002).

Whether the same individual gave a hissing display on different occasions was significantly repeatable (*F*_161,155_ = 1.70, *P *=* *0.0005, *R* [SE] = 0.27 [0.08]). Although the repeatability estimate was small, it was significant, implying that individuals tested multiple times had similar behavior more often than expected by chance.

Tits can either fly away from their nest box, or they can stay put and either perform the hissing display or not. Indeed, “fly-away” behavior and hissing behavior were not randomly associated (likelihood ratio χ^2^_1_ = 4.85, *P *=* *0.013). Among the 17 individuals that flew away, only one or 6% showed hissing display when present at the box on another occasion, whereas among the 296 individuals that did not fly away 28% showed the hissing display. Thus, among birds that reacted to the nest box visit there appeared to be 2 kinds of individuals, those that readily flew away, which rarely engaged in hissing, and those that stayed put and often hissed. Individuals that flew away produced on average 3.20 fledglings, whereas those that never flew away produced 4.38 fledglings (likelihood ratio χ^2^_1_ = 4.65, *P *=* *0.031).

The frequency of hissing display did not differ significantly between nest building, laying, incubation, and nestling periods (likelihood ratio χ^2^_3_ = 3.02, *P *=* *0.38). Thus there was no effect of the breeding stage on the probability of hissing.

### Hissing display and phenotypic quality of adults

**Figure 2.**
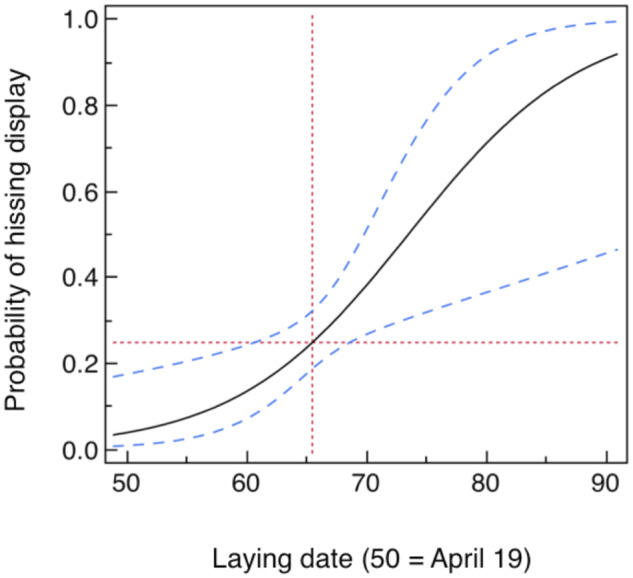
Probability of hissing display by adult female tits in relation to laying date in 224 nest boxes (50 = April 19). The blue lines are the 95% confidence intervals, whereas the red lines are located at the mean values.

Females emitting a hissing display laid eggs significantly later during the season than females without this behavior (Figure 2; likelihood ratio χ^2^_1_ = 8.48, *P *=* *0.0036, estimate [SE] = 0.139 [0.052]). This relationship was not confounded by species (great or blue tit: likelihood ratio χ^2^_4_ = 4.38, *P *=* *0.36) or age (likelihood ratio χ^2^_1_ = 0.11, *P *=* *0.74). Thus late breeding tits hissed on more than 90% of occasions, whereas the earliest tits hissed <10% of the occasions.

There was no significant difference between individuals with and without hissing behavior in terms of clutch size (likelihood ratio χ^2^_1_ = 0.03, *P *=* *0.86), number of fledglings (likelihood ratio χ^2^_1_ = 0.49, *P *=* *0.48), complete breeding failure (likelihood ratio χ^2^_1_ = 0.94, *P *=* *0.33), or breeding success (the proportion of eggs that resulted in fledglings: likelihood ratio χ^2^_1_ = 0.08, *P *=* *0.78).

### Hissing display and frequency-dependent selection

**Figure 3.**
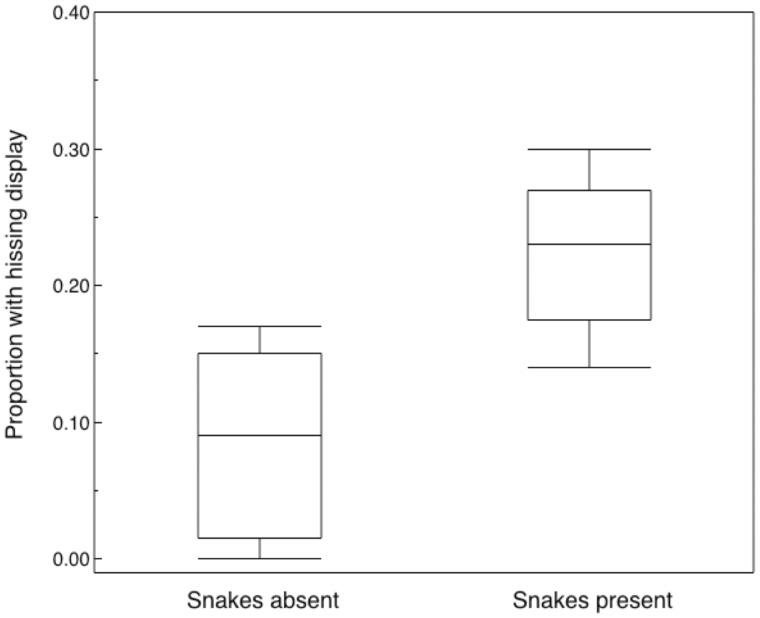
Box plots of the frequency of hissing display by adult female tits in 5 populations with and 5 populations without snakes in Denmark. Box plots present medians, quartiles, 5- and 95-percentiles, and extreme values.

If the hissing display is frequency-dependent, we would expect the frequency to increase in sites with higher abundance of snakes. That was the case for our study of the frequency of hissing display for 385 occupied nest boxes in 10 different sites in Denmark during 1972 and 2012–2013 (Figure 3; likelihood ratio χ^2^_1_ = 9.78, *P *=* *0.0018, estimate [SE] = 0.157 [0.054]). Thus hissing display was more common in areas with more snakes.

Next, we determined whether the frequency of hissing display in great tits was higher in China than in Denmark as predicted from a higher frequency of snakes in China. Although 27% of 313 tit individuals in Denmark in 2012–2013 hissed, 71% of 14 tits in China did so (likelihood ratio χ^2^_1_ = 9.74, *P *=* *0.0018). Snakes depredated most occupied nests in China (several boxes contained snakes at nest checks).

**Figure 4.**
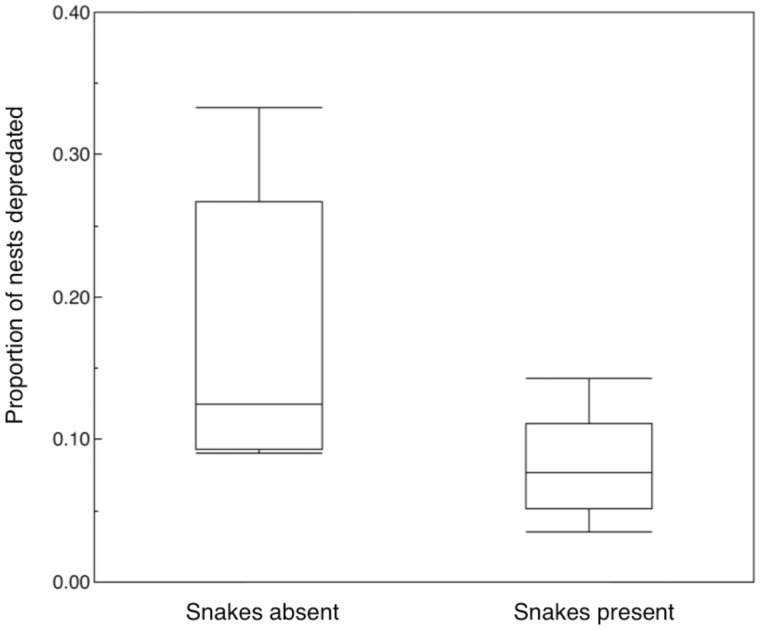
Proportion of nests depredated in 5 populations with and 5 populations without snakes in Denmark. Box plots present medians, quartiles, 5- and 95-percentiles and extreme values.

**Figure 5.**
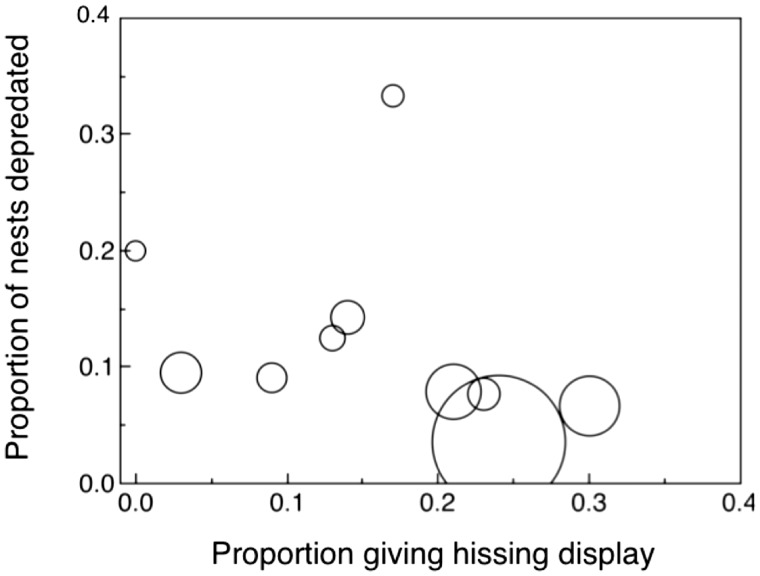
Proportion of nests depredated in relation to the proportion of individuals giving a hissing display in different populations. The size of circles reflects sample size.

The proportion of nests that were depredated was higher in Danish sites in 2012–2013 without snakes than in sites with snakes (Figure 4; likelihood ratio χ^2^_1_ = 9.78, *P *=* *0.0018, estimate [SE] = 0.157 [0.054]). Furthermore, the proportion of depredated nests decreased with increasing frequency of hissing display across sites in Denmark in 2012–2013 (Figure 5; likelihood ratio χ^2^_1_ = 9.78, *P *=* *0.0018, estimate [SE] = 0.157 [0.054]). This finding is consistent with the hypothesis that snake mimics are better protected in sites with more snake models.

## Discussion

Tits perform a snake-like display when inside their nest box by hissing at and thrusting their head toward any intruder including a human checking the contents of the nest box. Although we are aware of tits emitting a hissing sound when on the nest, we are still after >50 years of fieldwork startled and feel anxious when putting a hand into a nest box to check the nest contents. This suggests that humans also have an inherent snake aversion that can be exploited by birds. Recent studies have shown that pulvinar neurons are involved in rapid detection of snakes in humans (Le et al. 2013) providing a clear example of hard-wired anti-snake behavior. Although only a third of all tested birds gave the hissing display, it was repeatable among trials (see also Krams et al. 2014). Tits either flew away from their nest box, and only rarely gave the hissing display, or they stayed put and gave the display at a higher frequency. Hissing was more common among individuals breeding late during the season, when more snakes are out sunning, and in sites with a higher abundance of snakes. The hissing display was efficient at distracting predators because the frequency of nest predation was lower in sites with a higher frequency of hissing display. These observations are consistent with expectations for frequency-dependent mimicry.

Many species of snakes produce a stereotypic sound that is readily recognized as a snake hiss by many different animals including humans (e.g., Greene 1988; Young et al. 1999; Young 2003; Aubret and Mangin 2014). Cavity-nesting wrynecks, woodpeckers, owls, tits and warblers produce a hiss that mimics that of a snake when a potential predator disturbs a breeding bird inside the nest box (Burleigh 1930; Odum 1941; Steinfatt 1941; Hinde 1952; Löhrl 1964; Gompertz 1967; Warburton 1976; Dixon 1983; Klump and Shalter 1984). These 5 independent evolutionary events of snake-mimicry in the bird families Sylviidae, Paridae, Jyngidae, Picidae, and Strigidae appear to function as deterrents of predators. All these evolutionary events occurred in cavity-nesters or domed nesters perhaps because snakes are common inhabitants of cavities, and because cavity-nesting birds have few other possibilities of escape from a nest hole than by deterrence of a potential predator (Krams et al. 2014). We found evidence of convergence in the structure of hiss calls in tits toward the structure of hiss calls in snakes, and this convergence was specific to hiss calls and not to other calls such as contact calls of tits.

There were small differences in mean frequency and variance in hissing display between yearlings and older individuals with hissing being more common in yearlings. Such decreases with age may either be due to phenotypic plasticity or selective disappearance. Given the low level of repeatability, it seems likely that hissing behavior is conditional on personality or experience. This also suggests that it is not a fixed behavioral program and that it can be adjusted to local circumstances such as the frequency of predator visits. Alternatively, such differences between age classes may be due to bet hedging where organisms suffer decreased fitness among yearlings compared with increased fitness in stressful conditions among adults (Cohen 1966).

Frequency-dependent selection should result in an elevated frequency of snake mimicry in the presence of more frequent models. Indeed the efficiency of Batesian mimicry relies on confusion of the mimic with the model (Ruxton et al. 2004). Thus we would expect that the presence of more snakes in a site would allow for an elevated frequency of snake mimicry. This was indeed what we found across study sites differing in abundance of snakes. Spatial heterogeneity in the distribution of snake models and snake mimics can influence selection (Endler and Rojas 2009; Thorogood and Davies 2012) and facilitate the evolution of polymorphisms (Bleay et al. 2007; Comeault and Noonan 2011; Thorogood and Davies 2012). Distances of 5–70 km separated our study sites. Because birds with hissing display may disperse from one study site to another, such dispersal could reduce or eliminate any local adaptation to risk of nest predation. Great tits that are the most common tits in our study areas have a geometric mean natal dispersal distance of 0.80 km and a mean breeding dispersal distance of only 0.25 km (Paradis et al. 1998). Thus 70 km equals 88 natal dispersal distances (70 km/0.8 km). With nest predation rates of 10%, there should be strong selection against tits remaining on their nest in the absence of a hissing display. The most common predators on nests are martens *Martes foina*, domestic cats *Felis catus domesticus*, great spotted woodpeckers *Dendrocopus major* (mainly during winter), and wrynecks (see also Krams et al. 2014). Nest predators are likely to already have fully inherited fear of snakes from the remote ancestors of most terrestrial vertebrates, as shown by domestic cats being deterred from nest cavities following playback of hiss calls (Krams et al. 2014). In areas with no or few snakes, nest predators may be more prone to visit nest cavities and thus nest predation increases in areas with few snakes. Tits may, therefore, be less predisposed to hiss when nest predators are less likely to visit cavities. This alternative scenario is supported by the 2 “personalities” among tits with only aggressive individuals hissing, whereas less aggressive individuals fled their nest. This alternative hypothesis is not based on Batesian mimicry because it persists on the basis of the fixed behavior of predators, but not on the frequency of models. However, we consider that this alternative hypothesis is unlikely because the frequency of hissing is the highest in areas with more snakes, and the higher the abundance of snakes, the lower the risk of nest predation. If predators required direct experience with a dangerous model, as suggested by Speed and Turner (1999) and Ruxton et al. (2004), predators should not respond to hissing displays by leaving the mimic alone. Another possibility is that naïve predator individuals can learn from experienced individuals without suffering from the risk of imminent death caused by a model (Kendal et al. 2005; Davies and Welbergen 2009; Campobello and Sealy 2011). This seems unlikely given that the predators are solitary. A final possibility is that predator responses to hissing are hereditary and that the frequency of hissing display in different sites depends on the relative abundance of models and the fitness benefits from reduced rates of nest predation.

The relationships between hissing display and fitness components were generally weak. We found no relationship between hissing display and clutch size, brood size at hatching, or brood size at fledging. Likewise, there was no significant association between the age of females and hissing display. However, females that flew away from their nest box, when approached by a human, produced significantly fewer fledglings than did females that remained at their box. In addition, individuals that flew away, hissed on 6% of the cases, when present at the nest box, whereas individuals that stayed put hissed in 28% of the cases. Hence there was selection for tits to stay at the nest box and hiss. We found evidence of nest predation rate being elevated in study sites without snakes compared with sites with snakes (Figure 4), as we would expect if the effect of snake mimicry would be less efficient in the absence of snakes. Snakes are often involved in nest predation on tits in Europe (Hald-Mortensen 1970; Perrins 1979; Sorace et al. 2000). The negative association between the risk of nest predation and the presence of snakes that we found here is contrary to what would be expected if snakes were the nest predators. Indeed, there was a negative association between the frequency of nest predation and the frequency of hissing display across 10 study sites, as expected if hissing display was an efficient deterrent of nest predators.

In conclusion, the hissing display given by female tits during the breeding season constitutes snake mimicry, as it has converged toward the inhalation hiss of vipers and other snakes, thereby efficiently reduces the risk of nest predation. There was evidence of frequency-dependent mimicry because sites with more snakes had a higher frequency of mimics, and tits breeding at sites with a higher frequency of mimicry enjoyed a significant reduction in risk of nest predation.

## Funding 

This study was supported by the National Natural Science Foundation of China (Nos. 31772453 and 31970427 to W.L.). We would like to thank T. Su, J. Huo, and J. Wang for help with fieldwork.

## Supplementary Material

Supplementary material can be found at https://academic.oup.com/cz.

## Supplementary Material

zoaa028_Supplementary_Data
